# Antimicrobial Resistance Profile of Planktonic and Biofilm Cells of *Staphylococcus aureus* and Coagulase-Negative Staphylococci

**DOI:** 10.3390/ijms17091423

**Published:** 2016-09-01

**Authors:** Adilson de Oliveira, Valéria Cataneli Pereira, Luiza Pinheiro, Danilo Flávio Moraes Riboli, Katheryne Benini Martins, Maria de Lourdes Ribeiro de Souza da Cunha

**Affiliations:** 1 Departamento de Microbiologia e Imunologia, Instituto de Biociências, UNESP—Univ Estadual Paulista, Rua Professor Doutor Antonio Celso Wagner Zanin, s/n, Botucatu, São Paulo-CEP 18618-689, Brazil; adilson270193@gmail.com (A.d.O.); valeriacataneli@gmail.com (V.C.P.); luizapinheiro@ibb.unesp.br (L.P.); danilo.riboli@ibb.unesp.br (D.F.M.R.); katheryne_bm@yahoo.com.br (K.B.M.); 2 Departamento de Anatomia Patológica, Instituto Lauro de Souza Lima, Bauru 17034-971, Brazil

**Keywords:** *Staphylococcus aureus*, coagulase-negative staphylococci, biofilm, resistance, antimicrobials

## Abstract

The objective of the present study was to determine the antimicrobial resistance profile of planktonic and biofilm cells of *Staphylococcus aureus* and coagulase-negative staphylococci (CoNS). Two hundred *Staphylococcus* spp. strains were studied, including 50 *S. aureus* and 150 CoNS strains (50 *S*. *epidermidis*, 20 *S*. *haemolyticus*, 20 *S*. *warneri*, 20 *S*. *hominis*, 20 *S*. *lugdunensis*, and 20 *S. saprophyticus*). Biofilm formation was investigated by adherence to polystyrene plates. Positive strains were submitted to the broth microdilution method to determine the minimum inhibitory concentration (MIC) for planktonic and biofilm cells and the minimal bactericidal concentration for biofilm cells (MBCB). Forty-nine *Staphylococcus* spp. strains (14 *S. aureus*, 13 *S*. *epidermidis*, 13 *S. saprophyticus*, 3 *S*. *haemolyticus*, 1 *S*. *hominis*, 3 *S*. *warneri*, and 2 *S*. *lugdunensis*) were biofilm producers. These isolates were evaluated regarding their resistance profile. Determination of planktonic cell MIC identified three (21.4%) *S. aureus* strains that were resistant to oxacillin and six (42.8%) that were resistant to erythromycin. Among the CoNS, 31 (88.6%) strains were resistant to oxacillin, 14 (40%) to erythromycin, 18 (51.4%) to gentamicin, and 8 (22.8%) to sulfamethoxazole/trimethoprim. None of the planktonic isolates were resistant to vancomycin or linezolid. MICs were 2-, 4-, 8-, and up to 16-fold higher for biofilm cells than for planktonic cells. This observation was more common for vancomycin and erythromycin. The MBCB ranged from 8 to >256 µg/mL for oxacillin, 128 to >128 µg/mL for vancomycin, 256 to >256 µg/mL for erythromycin and gentamicin, >64 µg/mL for linezolid, and 32/608 to >32/608 µg/mL for sulfamethoxazole/trimethoprim. The results showed considerably higher MICs for *S. aureus* and CoNS biofilm cells compared to planktonic cells. Analysis of MBCM confirmed that even high concentrations of vancomycin were unable to eliminate the biofilms of *S. aureus* and CoNS species. Linezolid was the most effective drug in inhibiting staphylococci in the biofilm, without an increase in the MIC, when compared to planktonic cells. None of the isolates were resistant to this drug.

## 1. Introduction

*Staphylococcus aureus* and *Staphylococcus epidermidis* are common causative agents of infections associated with medical devices such as vascular catheters, prostheses, and artificial heart valves because of their capacity to adhere to the smooth surface of these materials and to form a biofilm [1]. In addition to *S. epidermidis*, other coagulase-negative staphylococci (CoNS) such as *S. hominis*, *S. haemolyticus*, *S. warneri*, *S. lugdunensis*, and *S. saprophyticus* have been associated with these infections. These microorganisms can also produce a biofilm, but at a lower frequency [2]. Biofilms are a group of sessile bacteria attached to a substrate and to each other, with each cell surrounded by an extracellular matrix [3,4]. In staphylococci, this matrix mainly consists of polysaccharide intercellular adhesin (PIA), but also contains proteins and extracellular DNA [5]. The biofilm prevents antimicrobial agents from entering the bacterium, thus interfering with the mechanism of action of these agents. In addition, biofilms protect bacteria from the host’s immune system, a fact leading to persistent infections that are difficult to treat [6].

The phenotype of bacteria growing in biofilms differs from that of planktonic cultures since the biofilm favors different bacterial interactions, increasing the frequency of mutations compared to planktonic cells and the transfer of resistance genes. Bacteria growing in biofilms can be up to 1000 times more resistant to antibiotic therapy than planktonic cells of the same microorganism [7].

Many studies have reported difficulties in treating biofilm-related infections with conventional antibiotic therapy. However, planktonic cells are still used in antimicrobial susceptibility tests performed in routine clinical laboratories for selection of the antibiotic. This fact impairs assessment of the efficacy of the antibiotic tested since in the patient these bacteria are protected by the biofilm and the response will not be the same as that obtained in the tests. The objective of the present study was to determine the susceptibility of *S. aureus* and CoNS planktonic and biofilm cells to six antimicrobial agents of different drug classes used to treat staphylococcal infections. 

## 2. Results

### 2.1. Investigation of Biofilm Production by a Modified Method of Adherence to Polystyrene Plates

**Figure 1:**
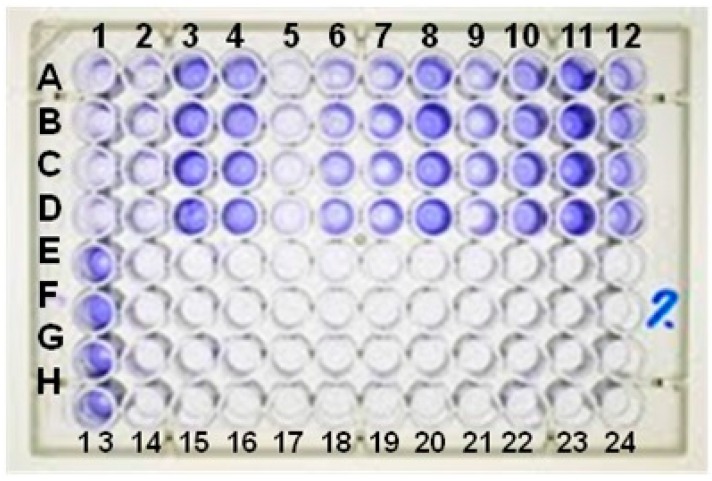
Polystyrene plate adherence test. A, B, C, and D represent four replicates of the same strain: columns 1, 2, 7, and 9 (weakly adherent); 3, 4, 8, 10, 11 (non-adherent), and 12 (strongly adherent). Rows E, F, G, and H represent four replicates of the same strain: columns 13 (*S. epidermidis* ATCC 35983—positive control), 14 (*S. epidermidis* ATCC 12228—negative control), and 15–22 (sterile Trypticase Soy Broth—TSB).

Analysis of the plates using a 540 nm filter (Figure 1) classified 41 (20.5%) of the 200 isolates as weakly adherent and 8 (4%) as strongly adherent, corresponding to 49 (24.5%) positive isolates and 151 (75.5%) negative non-adherent strains (Table 1).

### 2.2. Determination of the Minimum Inhibitory Concentration of Oxacillin, Vancomycin, Erythromycin, Gentamicin, Linezolid and Sulfamethoxazole/Trimethoprim for Staphylococcus spp. Planktonic Cells by the Broth Microdilution Method

Forty-nine staphylococcal isolates obtained from clinical materials of patients seen at the University Hospital of the Botucatu School of Medicine (Faculdade de Medicina de Botucatu—FMB), which were identified as biofilm producers by the phenotypic method (polystyrene plate adherence test), were analyzed. These isolates included 14 *S. aureus* and 35 CoNS (13 *S*. *epidermidis*, 13 *S. saprophyticus*, 3 *S*. *haemolyticus*, 1 *S*. *hominis*, 3 *S*. *warneri*, and 2 *S*. *lugdunensis*). Thirty-four (69.4%) of these isolates were resistant to oxacillin. None of the isolates were resistant to vancomycin. Twenty (40.8%) isolates were resistant to erythromycin and 15 (30.6%) exhibited intermediate resistance. Eighteen (36.7%) isolates were resistant to gentamicin and 1 (2%) exhibited intermediate resistance. None of the isolates were resistant to linezolid and 8 (16.3%) were resistant to sulfamethoxazole/trimethoprim. Table 2 lists the antibiotic-resistant strains according to species.

### 2.3. Determination of the Minimum Inhibitory Concentration of Oxacillin, Vancomycin, Erythromycin, Gentamicin, Linezolid, and Sulfamethoxazole/Trimethoprim for Staphylococcus spp. Biofilm Cells by the Broth Microdilution Method

The susceptibility of *Staphylococcus* spp. planktonic and biofilm cells to the six antimicrobial agents tested differed significantly between the two groups of cells. The minimum inhibitory concentrations (MICs) determined after 24 h are shown in Table 3.

Statistical analysis of the results revealed a significant increase in the resistance of *Staphylococcus* spp. biofilm cells compared to planktonic cells. Significant differences were observed for oxacillin in *S. epidermidis* (*p* = 0.042) and *S. saprophyticus* (*p* = 0.0098); for vancomycin in *S. aureus* (*p* = 0.0168), *S. epidermidis* (*p* = 0.0004), and *S. saprophyticus* (*p* ≤ 0.0001); for erythromycin in *S. aureus* (*p* = 0.0099), *S. haemolyticus* (*p* = 0.0301), *S. hominis* (*p* = 0.0056), *S. lugdunensis* (*p* = 0.0035), *S. saprophyticus* (*p* = 0.043), and *S. warneri* (*p* = 0.0005), and for gentamicin in *S. aureus* (*p* = 0.0124).

Regarding vancomycin in biofilm cells, 3 (6%) isolates became resistant and 15 (30.6%) developed intermediate resistance. In the case of the other drugs, there was a considerable increase in the number of susceptible planktonic strains that became resistant when growing in a biofilm, except for linezolid. None of the planktonic or biofilm strains were resistant to this drug (Table 4).

Analysis of the variations in MICs between planktonic and biofilm cells and of the percentage of resistance revealed significant differences for some species (Table 5). The MIC was considerably increased in biofilm cells of the isolates when compared to planktonic cells. A more than 8-fold increase was observed in some cases. Some isolates that were susceptible in the planktonic condition became resistant when growing in biofilms (Table 6). The minimal bactericidal concentration for biofilm cells (MBCB) was determined for the 49 biofilm-producing *Staphylococcus* spp. isolates. The results showed high MBCB of the drugs tested for all species, with most isolates being resistant to the antibiotics (Table 7).

## 3. Discussion

The use of antibiotics for the routine treatment of staphylococcal infections is generally able to eliminate planktonic cells, while biofilm cells can spread further even when treatment is interrupted since they remain protected from the action of the antimicrobial agents and from the host’s immune system [9]. This phenomenon is related to the presence of a large number of extracellular products such as polysaccharides and proteins, as well as adequate conditions for growth and expression of specific resistance genes [10].

A major issue in the antimicrobial resistance of staphylococci are oxacillin-resistant *S. aureus* because these microorganisms are generally resistant to multiple drugs and because of the large number of strains found. However, in the present study, only 3 (21.4%) of the 14 planktonic strains were resistant to oxacillin and 1 (2%) exhibited intermediate resistance to vancomycin and was resistant to oxacillin. In addition, 42.8% of the planktonic strains were resistant to erythromycin and susceptible to the remaining antibiotics tested. Studies have demonstrated a reduced effect of vancomycin and teicoplanin in *S. aureus* [11,12,13]. However, our data agree with the results reported by Menegotto and Picoli [14] who studied community *S. aureus* strains and found resistance to oxacillin among 7.5% of the isolates and to erythromycin among 45%, as well as full vancomycin susceptibility. Regarding CoNS, all *S. epidermidis* and *S. haemolyticus* isolates were resistant to oxacillin (100%) and most isolates were also resistant to erythromycin (77% and 100%, respectively), gentamicin (100% and 66.3%), and sulfamethoxazole/trimethoprim (61.5% and 33.3%). Similar results regarding resistance to these antimicrobial agents in the same species have been reported in other studies [14,15,16]. A significant percentage of *S. saprophyticus* isolates was also resistant to oxacillin (100%). In addition, 30.1% of the isolates were resistant to erythromycin, 23.0% to gentamicin, and 15.3% to sulfamethoxazole/trimethoprim. Our results on oxacillin resistance are consistent with those reported by Ferreira et al. [17]. Oxacillin resistance was also observed in the single *S. hominis* isolate of the present study and in one of the three *S. warneri* isolates. The two *S. lugdunensis* isolates were not resistant to any of the drugs. Similar results have been reported by Dundar et al. [18] and Frank et al. [19].

The presence of a biofilm increased the MIC 2-, 4-, 8-, and up to 16-fold compared to planktonic cells. This phenomenon was more common in the case of vancomycin and erythromycin. Antunes et al. [20] found up to a 64-fold increase in vancomycin MIC in *S. epidermidis* and *S. aureus*, strong biofilm producers. Other species such as *S. capitis* and *S. haemolyticus* exhibited small MIC variations in the presence or absence of a biofilm. In the present study, small MIC variations in the presence of a biofilm were observed for the *S. haemolyticus*, *S. hominis*, *S. warneri*, and *S. lugdunensis* isolates, probably because these species are weak biofilm producers [21] and because of the small number of isolates.

In contrast to the present study, Frank et al. [19] found *S. lugdunensis* to be resistant to linezolid and vancomycin in the presence of a biofilm. In our study, although the MIC of these drugs had increased subtly in the presence of a biofilm in *S. lugdunensis*, the isolates remained susceptible to vancomycin and linezolid. *Staphylococcus lugdunensis* is generally susceptible to most antimicrobial agents. The production of beta-lactamase is described in only 25% of the isolates and resistance to methicillin and glycopeptides is uncommon [21]. These observations demonstrate that biofilm production provides an important diffusion barrier that prevents the antimicrobial agent from reaching the bacterial cells [17].

This study determined the MBCB of *S. aureus* and of six CoNS species. The oxacillin MBCB ranged from 8 to >256 µg/mL in *S. aureus*, while the MBCB was >256 µg/mL in most *S. epidermidis*, *S. saprophyticus*, *S. haemolyticus*, *S. hominis*, and *S. lugdunensis* isolates. Only *S. warneri* exhibited a much lower oxacillin MBCB when compared to the other species, ranging from 8 to 32 µg/mL. In the study of Nabila et al. [21], the antibiotics exerted little effect on the viability of the intact and disrupted biofilms after detachment of the biofilm and recovery in culture medium, procedures similar to those used in this study. In the two studies (present one and the study of Nabila et al. [21]), even high doses of these antibiotics were not sufficient to eliminate *S. aureus* or *S. epidermidis* obtained from the biofilm. Sarginur et al. [22] also tested different antibiotics against *S. aureus* and *S. epidermidis* planktonic and biofilm cells and observed biofilm MBCB similar to those found in the present study for oxacillin, vancomycin, erythromycin, gentamicin, and linezolid. Another study testing different antibiotics against *S. lugdunensis* planktonic and biofilm cells found vancomycin and linezolid MBCM >128 µg/mL, values similar to those obtained in this study for *S. lugdunensis* (>128 and >64 µg/mL, respectively) [19].

Most studies in the literature investigating antimicrobial resistance in biofilms have used *S. aureus*, *S. epidermidis* and *S. lugdunensis*. The present study revealed that other species, which are less studied because of few reports of associated infections such as *S. haemolyticus*, *S. warneri*, and *S. hominis*, exhibited high MBCM of the drugs tested. For example, the vancomycin and linezolid MBCM were >128 and >64 µg/mL, respectively, suggesting that more attention should be paid to these CoNS species, which were also resistant to the antimicrobial agents especially when grown in biofilms.

Biofilm production is a successful strategy to guarantee microbial survival and to establish infection. Since host defense mechanisms and the response to antimicrobial agents are compromised by the presence of bacterial biofilms, biofilm-related chronic infections and sepsis are a matter of great concern. Consequently, different protocols for profiling antimicrobial resistance should be implemented in clinical microbiology laboratories to permit the selection of appropriate treatment.

## 4. Materials and Methods

### 4.1. Strains

Two hundred *Staphylococcus* spp. strains, 50 *S. aureus* isolates and 150 CoNS isolates, were studied. The CoNS strains included 50 *S*. *epidermidis*, 20 *S*. *haemolyticus*, 20 *S*. *warneri*, 20 *S*. *hominis*, and 3 *S*. *lugdunensis* strains isolated from blood cultures and 20 *S. saprophyticus* isolated from the urine of patients with urinary tract infection seen at the University Hospital of FMB, Unesp, São Paulo, Brazil. The present study was approved by Committee for Ethics in Research (“Comitê de Ética em Pesquisa” from “Faculdade de Medicina de Botucatu”, Botucatu, São Paulo State, Brazil—Protocol 3783–2011). In view of the difficulty of isolating *S. lugdunensis* from human clinical samples, 13 *S. lugdunensis* strains isolated from milk samples of goats with mastitis were included. The following international reference strains were used: *S. epidermidis* ATCC 12228 and *S. aureus* ATCC 33591 (negative control), and *S. epidermidis* ATCC 35983 and *S. aureus* ATCC 29213 (positive control). 

### 4.2. Identification of the Microorganisms

After growth on blood agar plates, the microorganisms were submitted to Gram staining for analysis of purity of the strain, morphology, and specific staining. The microorganisms were identified according to Koneman et al. [23].

### 4.3. Identification of Bacteria of the Genus Staphylococcus

The tube coagulase test was used for the identification of *S. aureus*. CoNS were identified according to the criteria proposed by Cunha et al. [24] using a simplified scheme of biochemical tests: catalase, coagulase, sugar utilization (xylose, sucrose, trehalose, mannitol, and maltose), characterization of hemolysins, nitrate reduction, urease, ornithine decarboxylase, and novobiocin susceptibility. Next, the CoNS species were genotypically confirmed by the internal transcribed spacer-PCR (ITS-PCR) method described by Couto et al. [25] using primers that target conserved sequences adjacent to the 16S and 23S genes.

### 4.4. Investigation of Biofilm Production by a Modified Method of Adherence to Polystyrene Plates 

The method of biofilm production on culture plates proposed by Christensen et al. [26] was used, with some modifications according to Oliveira et al. [27]. Weakly adherent was defined as an optical density higher than the cutoff point or equal to or lower than double the cutoff point, and strongly adherent as an optical density higher than double the cutoff point.

### 4.5. Determination of the Minimum Inhibitory Concentration of Oxacillin, Vancomycin, Erythromycin, Gentamicin, Linezolid, and Sulfamethoxazole/Trimethoprim for Staphylococcus spp. Planktonic Cells by the Broth Microdilution Method

For the broth microdilution method, sterile microtiter plates containing cation-adjusted Mueller–Hinton broth (Oxoid, UK) as recommended by the Clinical and Laboratory Standards Institute (CLSI) [8] were used. A stock solution of each drug was prepared in distilled water at a concentration of 3200 µg/mL. In a microtiter plate, serial dilutions were prepared in Mueller–Hinton broth on a logarithmic scale of 2 [8], ranging from 0.125 to 128 µg/mL, in a final volume of 100 µL. For preparation of the inoculum, the strains were first seeded onto blood agar and incubated for 24 h. After this period, the isolated colonies were transferred to Brain Heart Infusion (BHI) broth and incubated. A bacterial suspension with a turbidity corresponding to 0.5 McFarland standard (1 × 10^8^ CFU/mL) was prepared and diluted 1:1000. Next, 200 µL of this suspension was added to the wells, corresponding to a final bacterial concentration of 5 × 10^4^. The plates were incubated in an oven at 35 °C and the minimum inhibitory concentration (MIC) was determined after 24 and 48 h. A positive control containing broth and the bacterial suspension and a negative control containing only Mueller–Hinton broth were included. Additionally, *Enterococcus faecalis* ATCC 29212 and *S. aureus* ATCC 29213, which are susceptible to vancomycin, were used as negative controls. Vancomycin-resistant *E. faecalis* ATCC 51299 and oxacillin-resistant *S. aureus* ATCC 33591 served as positive controls. The MIC was defined as the lowest concentration of the antibiotic that completely inhibited the growth of the microorganism as detected by the naked eye. Wells showing turbidity and/or the presence of bacteria on the bottom of the well were classified as positive growth [8]. The classification of the MICs into susceptible, intermediate, and resistant was based on the breakpoints and definitions of the CLSI [8].

### 4.6. Determination of the Minimal Bactericidal Concentration of Oxacillin, Vancomycin, Erythromycin, Gentamicin, Linezolid, and Sulfamethoxazole/Trimethoprim for Staphylococcus spp. Biofilm Cells by the Broth Microdilution Method

**Figure 2:**
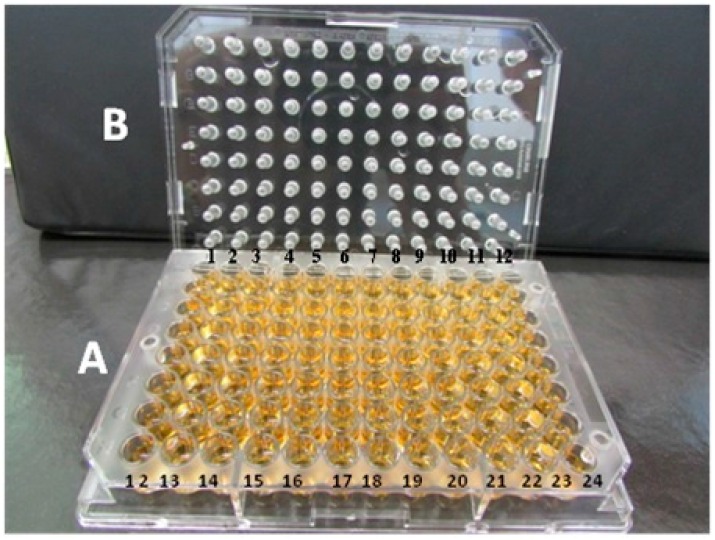
(**A**) Plate containing TSB with 2% glucose used for biofilm formation, cell recovery, and determination of MIC and MBCB; (**B**) Lid with pins for formation of staphylococcal biofilms.

The minimal bactericidal concentrations for biofilm cells (MBCB) were determined using an adaptation of a previously published method [19]. The isolates cultured for 22 h in Trypticase Soy Broth (TSB) plus 2% glucose were adjusted to a 1.0 McFarland standard (corresponding to 1 to 2 × 10^8^ CFU/mL) and diluted 1:50 in TSB-2% glucose. Aliquots (200 µL) were transferred to 96-well flat bottom plates (Nunclon Delta, Nunc, Roskilde, Denmark) covered with a 96-pin lid (Nunc™-ImmunoTSP; Nunc) and incubated for 24 h to permit the formation of a biofilm on the pins (Figure 2). For removal of non-adherent cells, the films formed on the pins were washed by immersion in a series of three 96-well plates filled with 200 µL sterile phosphate-buffered saline (PBS). The lid with the pins was placed on flat-bottom plate prepared for susceptibility testing by broth microdilution. The wells contained 200 µL of the antibiotic diluted in cation-supplemented Mueller–Hinton broth (CAMHB; 100 mg/mL calcium and 50 mg/mL magnesium) or 200 µL CAMHB without the drug for the control of positive growth. The biofilms were exposed to the antibiotics for 24 h. After this period, the lid with the pins was removed, washed three times in PBS as described above, and placed in 96 wells containing 200 µL TSB-2% glucose. On that occasion, before discarding the plate with the antibiotics, growth was evaluated by the naked eye to determine the MIC of the antibiotics for biofilm cells. The biofilm cells formed on the pins were dislodged by sonication (Hielscher Ultrasound Technology, Teltow, Germany, UIP250MTP) at 40 kHz for 5 min in 96-well plates containing fresh culture medium for recovery of the cells. The lid with the pins was discarded and replaced with a normal lid and the optical density was measured in a plate reader equipped with a 600 nm filter. Wells containing pure TSB-2% glucose (without inoculum) were used as spectrophotometric controls of sterility. The plate was incubated for 24 h and a second measurement of optical density was obtained at 600 nm. The MBCB was defined as the lowest concentration of the drug that resulted in a 10% change in optical density at 600 nm compared to the reading obtained for the positive control growth between readings performed before incubation and after 24 h [28].

### 4.7. Statistical Analysis

The MIC between *Staphylococcus* spp. planktonic and biofilm cells were analyzed using the *Chi*-squared or Fisher’s exact tests, adopting a level of significance of <0.05.

## Figures and Tables

**Table 1:** Evaluation of biofilm production of the staphylococcal species by the phenotypic method.

Species	Number of Isolates	Plate Test
*S. aureus*	13	WA
*S. aureus*	1	SA
*S. aureus*	36	NA
*S. epidermidis*	9	WA
*S. epidermidis*	4	SA
*S. epidermidis*	37	NA
*S. saprophyticus*	12	WA
*S. saprophyticus*	1	SA
*S. saprophyticus*	7	NA
*S. haemolyticus*	2	WA
*S. haemolyticus*	1	SA
*S. haemolyticus*	17	NA
*S. hominis*	1	WA
*S. hominis*	19	NA
*S. warneri*	3	WA
*S. warneri*	17	NA
*S. lugdunensis*	1	WA
*S. lugdunensis*	1	SA
*S. lugdunensis*	18	NA

WA: weakly adherent; SA: strongly adherent; NA: non-adherent.

**Table 2:** Species distribution of antimicrobial resistance among planktonic bacteria.

Microorganisms	OXA		VAN		ERY		GEN		LIN		SX-TR	
Species	R	IR	R	IR	R	IR	RN	IR	R	IR	R	IR
	N	N	N	N	N	N	N	N	N	N	N	N
*S. aureus* (*n* = 14)	3	●	0	0	6	5	0	1	0	●	0	●
*S*. *epidermidis* (*n* = 14)	13	●	0	0	10	0	12	0	0	●	5	●
*S. saprophyticus* (*n* = 14)	13	●	0	0	4	4	3	0	0	●	2	●
*S*. *haemolyticus* (*n* = 3)	3	●	0	0	0	2	2	0	0	●	1	●
*S*. *hominis* (*n* = 1)	1	●	0	0	0	1	1	0	0	●	0	●
*S*. *warneri* (*n* = 3)	1	●	0	0	0	3	0	0	0	●	0	●
*S*. *lugdunensis* (*n* = 2)	0	●	0	0	0	0	0	0	0	●	0	●
Total	34	-	0	0	20	15	18	1	0	0	8	-

R: resistant; IR: intermediate resistance; ●: drug without MIC of intermediate resistance; OXA: oxacillin; VAN: vancomycin; ERY: erythromycin; GEN: gentamicin; LIN: linezolid; SX-TR: sulfamethoxazole/trimethoprim; N: Number of strains.

**Table 3:** Susceptibility of *Staphylococcus* spp. planktonic and biofilm cells (*n* = 49).

Quantification	Antibiotic (µg/mL)					
	OXA	VAN	ERY	GEN	LIN	SX-TR
MIC_50_	2	2	2	2	1	0.5/9.5
MIC_90_	64	4	>128	64	2	8/152
MIC variation	<0.125–>128	1–4	0.25–>128	0.125–>128	0.25–2	0.03/0.59–16/304
MIC*_50_	4	4	32	4	1	<0.5/9.5
MIC*_90_	128	8	256	256	2	8/152
MIC variation	32–256	1–32	0.25–256	0.25–256	0.25–4	<0.5/9.5–16/304

MIC: minimum inhibitory concentration for planktonic cells; MIC*: minimum inhibitory concentration for biofilm cells. OXA: oxacillin; VAN: vancomycin; ERY: erythromycin; GEN: gentamicin; LIN: linezolid; SX-TR: sulfamethoxazole/trimethoprim.

**Table 4:** Comparison of the antibiotic resistance profile between *Staphylococcus* spp. planktonic and biofilm cells.

Antibiotic	Planktonic Cells		Biofilm Cells	
Drugs	R	IR	R	IR
	N (%)	N (%)	N (%)	N (%)
Oxacillin	34 (69.4)	●	38 (77.6)	●
Vancomycin	0 (0)	1 (2)	3 (6.1)	15 (30.6)
Erythromycin	20 (40.8)	15 (30.6)	33 (67.3)	9 (18.4)
Gentamicin	18 (36.7)	1 (2)	21 (42.8)	0 (0)
Linezolid	0 (0)	●	0 (0)	●
SX-TR	8 (16.3)	●	11 (22.4)	●

●: drug without MIC of intermediate resistance; R: resistant; IR: intermediate resistance; SX-TR: sulfamethoxazole/trimethoprim; N: Number of strains.

**Table 5:** Comparison of minimum inhibitory concentration and percentage of resistance between planktonic and biofilm cells of *Staphylococcus aureus* and coagulase-negative staphylococci.

Species	Oxacillin				Vancomycin				Erythromycin				Gentamicin				Linezolid				Sulfamethoxazole/Trimethoprim			
	MIC		MIC *		MIC		MIC*		MIC		MIC *		MIC		MIC*		MIC		MIC*		MIC		MIC *	
Organism (N)	Var.	% R	Var.	% R	Var.	% R	Var.	% R	Var.	% R	Var.	% R	Var.	% R	Var.	% R	Var.	% R	Var.	% R	Var.	% R	Var.	% R
*S. aureus* (14)	0.5–128	21.4	0.5–128	42.9	1-4	2 **	2–4	35.7 *	0.5–128	11 **	0.5–256	11 **	1–8	7.1 **	1–32	21.4 **	0.5-1	0	0.5–1	0	0.03/0.59–0.25/4.75	0	<0.5/9.5	0
*S. epidermidis* (13)	0.5–64	100	0.5–256	100	2–4	0	2–32	38.5*	0.25–256	77	0.25–256	77	0.5–256	92.3	4–256	100 **	0.25–1	0	0.25–1	0	<0.5/9.5–8/152	38.5	<0.5/9.5–8/152	61.5
*S. saprophyticus* (13)	1–4	100	2–16	100	2–4	0	2–32	53.8	0.5–256	61.3	0.5–256	92.3	0.125–32	23	0.25–64	23	1–2	0	1–4	0	0.125–2.38	15.4	<0.5/9.5–16/304	15.4
*S. haemolyticus* (3)	2–256	100	4–256	100	2	0	2–4	0	0.25–2	33.3 **	1–16	100 **	1–32	66.6	2–64	66.6	0.25–0.5	0	0.25–0.5	0	0.5/4.75–16/304	33.3	0.5/4.75–16/304	33.3
*S. hominis* (1)	256	100	256	100	4	0	4	0	4	100 *	256	100	32	100	64	100	1	0	1	0	1/19	0	1/19	0
*S. lugdunensis* (2)	0.5–1	0	1–2	0	1	0	1–2	0	0.25–8	50	16–256	100	2	0	4	0	0.5	0	0.5–1	0	0.5/9.5	0	0.5/9.5–1/19	0
*S. warneri* (3)	0.25–16	33.3	0.25–16	66.6	1	0	1–8	33.3 *	1–2	33.3*	4-16	100 **	0.25–0.5	0	0.5-1	0	0.25–1	0	0.25—1	0	0.06/1.18–0.5/9.5	0	<0.5/9.5	0

N: Number of strains; MIC: minimum inhibitory concentration for planktonic cells; MIC*: minimum inhibitory concentration for biofilm cells; Var.: variation in MIC; % R: percentage of resistance; * Intermediate resistance; ** intermediate and total resistance. Susceptibility/resistance cut-offs according to the CLSI [8].

**Table 6:** Variation in the increase of minimum inhibitory concentrations and change from the susceptible to resistant category in planktonic and biofilm cells.

Species	Oxacillin				Vancomycin				Erythromycin				Gentamicin				Linezolid				Sulfamethoxazole/Trimethoprim			
Organism (N)	2X	4X	8X *	S-R	2X	4X	8X *	S-R	2X	4X	8X *	S-R	2X	4X	8X *	S-R	2X	4X	8X *	S-R	2X	4X	8X *	S-R
*S. aureus* (14)	21.4	0	14.2	21.4	50	7.1	0	28.6	14.3	14.3	35.7	0	21.4	0	14.3	14.8	7.1	0	0	0	21.4	0	0	0
*S. epidermidis* (13)	35.7	15.3	7.7	0	23	23	7.7	46.1	38.5	7.7	0	0	7.7	7.7	0	0	38.7	0	0	0	30.8	7.7	0	23
*S. saprophyticus* (13)	38.4	7.7	7.7	0	23	38.4	15.4	53.8	23	7.7	38.4	30.8	61.5	0	0	0	7.7	0	0	0	15.4	0	0	0
*S. haemolyticus* (3)	33.3	0	0	0	33.3	0	0	0	0	66.6	33.3	33.3	100	0	0	0	33.3	0	0	0	0	0	0	0
*S. hominis* (1)	0	0	0	0	0	0	0	0	0	0	100	100	100	0	0	0	0	0	0	0	0	0	0	0
*S. lugdunensis* (2)	100	0	0	0	50	0	0	0	0	0	100	100	0	0	0	0	50	0	0	0	50	0	0	0
*S. warneri* (3)	0	0	33.3	33.3	33.3	0	33.3	33.3	0	33.3	66.6	66.6	66.6	33.3	0	0	0	0	0	0	0	0	0	0

N: Number of strains; 2X: Percentage of isolates with a 2-fold increase in MIC in the biofilm; 4X: percentage of isolates with a 4-fold increase in MIC in the biofilm; 8X *: percentage of isolates with at least an 8-fold in MIC in the biofilm; S-R: percentage of isolates that are resistant or intermediate resistant only in the presence of a biofilm.

**Table 7:** Profile of minimal bactericidal concentration (µg/mL) for biofilm cells of *Staphylococcus aureus* and coagulase-negative staphylococci.

Organism (N)	OXA		VAN		ERY		GEN		LIN		SX-TR	
	N (%)	MBCB	N (%)	MBCB	N (%)	MBCB	N (%)	MBCB	N (%)	MBCB	N (%)	MBCB
*S. aureus*(14)	1 (7.1) 1 (7.1) 1 (7.1) 2 (14.2) 9 (64.3)	8 64 128 256 >256	2 (14.2) 12 (85.7)	128 >128	1 (7.1) 13 (92.9)	256 >256	1 (7.1) 13 (92.9)	256 >256	14 (100)	>64	14 (100)	>32/608
*S. epidermidis*(13)	2 (15.4) 11 (84.6)	256 >256	1 (7.7) 12 (92.3)	128 >128	3 (23.1) 10 (76.9)	256 >256	3 (23.0) 10 (77.0)	256 >256	13 (100)	>64	1 (7.7) 12 (92.3)	32/608 >32/608
*S. saprophyticus* (13)	1 (7.7) 1 (7.7) 11 (84.6)	128 256 >256	1 (7.7) 12 (92.3)	128 >128	3 (23.1) 10 (76.9)	256 >256	9 (69.2) 4 (30.8)	256 >256	13 (100)	>64	2 (1543) 11 (84.6)	32/608 >32/608
*S. haemolyticus*(3)	3 (100)	>256	1 (33.3) 2 (66.7)	128 >128	1 (33.3) 2 (66.7)	256 >256	1 (33.3) 2 (66.7)	256 >256	3 (100)	>64	3 (100)	>32/608
*S. hominis*(1)	1 (100)	>256	1 (100)	>128	1 (100)	>256	1 (100)	>256	1 (100)	>64	1 (100)	>32/608
*S. warneri* (3)	1 (33.3) 1 (33.3) 1 (33.3)	8 16 32	3 (100)	>128	1 (33.3) 2 (66.7)	256 >256	1 (33.3) 2 (66.7)	256 >256	1 (33.3) 2 (66.7)	64 >64	3 (100)	>32/608
*S. lugdunensis* (2)	2 (100)	>256	2 (100)	>128	2 (100)	>256	2 (100)	256	2 (100)	>64	2 (100)	>32/608

N: Number of strains; MBCB: minimal bactericidal concentration for biofilm cells; OXA: oxacillin; VAN: vancomycin; ERY: erythromycin; GEN: gentamicin; LIN: linezolid; SX-TR: sulfamethoxazole/trimethoprim.
